# Involvement of M1/M2 Macrophage Polarization in Reparative Dentin Formation

**DOI:** 10.3390/life12111812

**Published:** 2022-11-07

**Authors:** Masataka Kadowaki, Shinichiro Yoshida, Tomohiro Itoyama, Atsushi Tomokiyo, Sayuri Hamano, Daigaku Hasegawa, Hideki Sugii, Hiroshi Kaneko, Risa Sugiura, Hidefumi Maeda

**Affiliations:** 1 Department of Endodontology and Operative Dentistry, Faculty of Dental Science, Kyushu University, 3-1-1 Maidashi, Higashi-ku, Fukuoka 812-8582, Japan; 2 Department of Endodontology, Kyushu University Hospital, Kyushu University, 3-1-1 Maidashi, Higashi-ku, Fukuoka 812-8582, Japan; 3 Faculty of Dental Science, OBT Research Center, Kyushu University, 3-1-1 Maidashi, Higashi-ku, Fukuoka 812-8582, Japan

**Keywords:** macrophage, direct pulp capping, dental pulp stem cell, reparative dentin formation, odontoblastic differentiation

## Abstract

In cases in which dental pulp tissue is accidentally exposed, direct pulp capping is often performed to induce reparative dentin formation. Although macrophages are essential for the inflammatory response and tissue repair, the emergence pattern and the role of macrophages in dental pulp tissue have not been clarified. Here, we investigated the emergence of M1/M2 macrophages in dental pulp tissue after a direct pulp capping and the effects of M2 macrophages on odontoblastic differentiation of the dental pulp stem cell (DPSC) clones. The emergence of macrophages in dental pulp tissue was investigated using a rat direct pulp capping model. Alizarin Red S staining and quantitative RT-PCR was performed to examine the effect of M2 macrophages on the mineralization and odontoblastic differentiation of DPSC clones. Immunohistochemical staining revealed that M1 macrophages were detected in dental pulp tissue after treatment and increased in number at three days after treatment. However, M2 macrophages gradually increased in number in dental pulp tissue after treatment, with the highest level recorded at seven days post-operation. Additionally, conditioned medium from M2 macrophages induced odontoblast-like differentiation of DPSC clones. These results suggest that macrophages play a role in the inflammatory response and reparative dentin formation after dental pulp exposure.

## 1. Introduction

Dental caries, traumatic injuries or dental treatment sometimes cause dentin defects and result in dental pulp exposure. Once dental pulp tissue is exposed, various kinds of growth factors or cytokines from inflammatory cells activate the dental pulp stem cells (DPSCs) or progenitors to differentiate into odontoblasts to induce reparative dentin at the exposure site [1]. However, in severe cases of dental pulp exposure or bacterial infection, endodontic treatment is needed to remove the inflamed and/or infected pulp tissue. It has been reported that endodontically-treated teeth are at greater risk of root fracture, compared with teeth with healthy dental pulp tissue [2]. Thus, the preservation of dental pulp tissue by direct pulp capping treatment is worthwhile to lengthen the lifespan of the teeth and could be a great contribution to improve public health [3]. As a direct pulp capping agent, mineral trioxide aggregate (MTA) is a first-choice material, based on the histological and clinical evidence that it induces reparative dentin at pulp exposure sites, which the mechanisms involved in it have not been elucidated [4,5,6].

Macrophages play indispensable roles in the inflammatory response and regeneration of numerous tissues [7]. Macrophages are divided into three phenotypes: unpolarized M0, M1 and M2 macrophages [8,9]. M0 macrophages polarize toward M1 macrophages in response to interferon gamma (IFN-γ) [10] produced by helper T lymphocytes type 1, and bacterial lipopolysaccharide (LPS) [11]. M1 macrophages show an inflammatory response against pathogens and foreign materials by secreting various pro-inflammatory cytokines or toxic effector molecules, such as interleukin 1β (1L-1β), tumor necrosis factor alpha (TNF-α), IL-6, [12] reactive oxygen species, and nitric oxide [13]. Moreover, M2 macrophages produce anti-inflammatory factors, such as transforming the growth factor beta (TGF-β) [14], IL-10 [15], and the angiogenic vascular endothelial growth factor [16], which contribute to the wound healing of various tissues, such as bone and skin [17,18,19]. However, the emergence of macrophages in injured dental pulp tissue and their role in the regenerative process after direct pulp capping remain unclear.

Therefore, this study aimed to investigate the emergence of macrophages in dental pulp tissue after direct pulp capping, and the effect of M2 macrophages on the odontoblastic differentiation of DPSC clones.

## 2. Materials and Methods

### 2.1. Induction of M0 Macrophages into M1 and M2 Macrophages

THP-1 (JCRB0112), a cell line of human monocyte, was purchased from the JCRB cell bank (Osaka, Japan) and cultured in RPMI 1640 (Nacalai Tesque, Kyoto, Japan), including 10% fetal bovine serum (FBS; Sigma-Aldrich, St. Louis, MO, USA) and 100 U/mL penicillin G (Gibco-BRL, Grand Island, NY, USA). For the M0 macrophage induction, THP-1 cells were stimulated with 10^−7^ M phorbol 12-myristate 13-acetate (PMA; Sigma-Aldrich) for one day. Following the PMA-treatment, the medium was replaced with RPMI1640 with 10% FBS and maintained for 24 h. Then, the M0 macrophages were further treated with 40 ng/mL LPS (Sigma-Aldrich) and 100 ng/mL IFN-γ (Pepro-Tech, Rocky Hill, NJ, USA) or 40 ng/mL IL-4 (Pepro-Tech, Rocky Hill, NJ, USA) for two days to differentiate into M1 or M2 macrophages, respectively.

### 2.2. Preparation of the Conditioned Medium from the M2 Macrophages

Following the washing with PBS, M2 macrophages were maintained in α-MEM for 24 h. The conditioned medium from M2 macrophages (M2-CM) was collected using a 0.22 µm filter (MilliporeSigma, Darmstadt, Germany) and stored at 4 °C.

### 2.3. Cell Isolation and Maintenance 

Human DPSC clones were isolated from a healthy premolar that was extracted from a 22-year-old female at Kyushu University Hospital, for orthodontic reasons, as we reported previously [20,21]. Briefly, the dental pulp tissue was incubated with 0.25% trypsin and 0.2% collagenase (FUJIFILM Wako Pure Chemical Industries Ltd., Osaka, Japan) in α-minimal essential medium (α-MEM; Gibco-BRL) at 37 °C for 20 min. The DPSC clones were isolated by a single colony selection from the dissociated cells and maintained in α-MEM, including 10% FBS (10% FBS/α-MEM). All procedures comply with the regulation of the Research Ethics Committee, Kyushu University (approval number: 20A-3).

### 2.4. Odontoblastic Differentiation

The DPSC clones were cultured in 10% FBS/α-MEM containing 1 mM CaCl_2_ (FUJIFILM Wako Pure Chemical Industries Ltd., Osaka, Japan) (differentiation medium [DM]) [22,23] with or without M2-CM at concentrations of 5, 10, 25, or 50% using 24-well plates (Becton Dickinson Labware, Lincoln Park, NJ, USA) for four weeks with medium changes every other day. The cells were then formalin-fixed (FUJIFILM Wako Pure Chemical Industries Ltd., Osaka, Japan) for Alizarin Red S (Sigma-Aldrich, Burlington, MA, USA) staining. BZ-9000 fluorescence microscope (Keyence Corporation, Osaka, Japan) and MZ-II-software (Keyence Corporation, Osaka, Japan) were used to identify the positive staining. The total RNA from the cells of five days of culture was isolated by a phenol/chloroform method [24] using TRIzol Reagent (Invitrogen, Carlsbad, CA, USA) and chloroform (Nacalai Tesque, Kyoto, Japan), followed by alcohol precipitation.

### 2.5. Quantitative RT-PCR

The purity and the concentration of the total RNA was measured using a NanoDrop Lite Spectrophotometer (Thermo Fisher Scientific Inc., Walthum, MA, USA). First-strand complementary DNA was synthesized from 1 mg total RNA using an ExScript RT Reagent kit (Takara Bio Inc., Shiga, Japan). The mRNA expressions of target genes were evaluated by quantitative RT-PCR using a SYBR Green II RT-PCR kit (Takara Bio Inc., Shiga, Japan) in a Thermal Cycler Dice Real Time System (Takara Bio Inc., Shiga, Japan), as reported previously [21]. The primer sequences were as follows: DSPP (129 bp) (forward 5′-CCCTGAAGGCAAAGAAGATCCC-3′, reverse 5′-TGGTTGAGCTTCTGGGTGTCC-3′), DMP-1 (200 bp) (forward 5′-AGACACTGGCCTCAGCCAAC-3′, reverse 5′-CGGGGTTATCTCCCCTGGAC-3′), BMP2 (74 bp) (forward 5′-TCCACTAATCATGCCATTGTTCAGA-3′, reverse 5′-GGGACACAGCATGCCTTAGGA-3′), and human β-actin (189 bp) (forward 5′-TGGCACCCAGCACAATGAA-3′, reverse 5′-CTAAGTCATAGTCCGCCTAGAAGCA-3′). To calculate the relative mRNA expression, ΔΔCt values were applied using β-actin as an internal calibrator.

### 2.6. Direct Pulp Capping Model

The procedure has conducted, according to our previous reports [20,21]. Eighteen eight-week-old male Wistar rats (Kyudo, Saga, Japan) were used in this experiment and anesthesia, including 0.15 mg/kg of medetomidine hydrochloride (Kyoritsu Seiyaku, Tokyo, Japan), 2 mg/kg of midazolam (Sandoz, Tokyo, Japan), and 2.5 mg/kg of butorphanol tartrate (Meiji Seika Pharma, Tokyo, Japan) was performed by intraperitoneal injection. The access cavity was created with a #1/2 round steel bur (Dentsply Maillefer, Tienen, Belgium) on the occlusal surface of the upper left first molar, and pulp was exposed using a sterile dental explorer. Direct pulp capping was performed with MTA cement (ProRoot, Dentsply Sirona, Charlotte, NC, USA), and covered with glass ionomer cement (Fuji IX, GC Corporation, Tokyo, Japan). The upper right first molar of the same animal served as a control. The animals were transcardially perfused with 4% paraformaldehyde (PFA; Nacalai Tesque) at 1, 2, 3, 5, 7, and 14 days after treatment. The tissues were collected and decalcified using Kalkitox (FUJIFILM Wako Pure Chemical Industries Ltd., Osaka, Japan) for 48 h and embedded in paraffin. Reparative dentin formation was confirmed by Hematoxylin-eosin (H-E) staining. All procedures comply with the regulations of the Animal Ethics Committee of Kyushu University (approval number: A20-210-0).

### 2.7. Staining Procedures

Samples were deparaffinized and subjected to immunological staining. The blocking procedure was performed using 2% bovine serum albumin (BSA; Nacalai Tesque, Kyoto, Japan) for 1 h at room temperature and the sections were incubated overnight with primary antibodies at 4 °C. Then, the samples were further subjected to a biotinylated anti-rabbit secondary antibody (426011, Nichirei Biosciences Inc., Tokyo, Japan) treatment, followed by avidin-peroxidase conjugate (426061, Nichirei Biosciences Inc., Tokyo, Japan). The immuno-positive reactions were identified with diaminobenzidine (415171, Nichirei Biosciences Inc., Tokyo, Japan), and counterstained with hematoxylin (FUJIFILM Wako Pure Chemical Industries Ltd., Osaka, Japan). Rabbit polyclonal anti-CD86 antibody (NBP2-67417, NOVUS Biologicals, Centennial, CO, USA; 1:50), rabbit polyclonal anti-CD206 antibody (ab64693, Abcam, Cambridge, MA, USA; 1:10,000), or normal rabbit immunoglobulin G (IgG) (2729, Cell Signaling Technology, Beverly, MA, USA) were used in this experiment. The images were taken using a BX41 microscope (Olympus Medical, Tokyo, Japan).

Immunofluorescence staining was performed, as we reported previously [22]. Briefly, after the fixation of macrophages with 4% PFA (Nacalai Tesque) for 15 min, the blocking procedure was performed, as described above. The macrophages were then subjected to rabbit polyclonal anti-CD80 antibody (BS-2211R, Bioss Antibodies Inc., Woburn, MA, USA; 1:50), rabbit polyclonal anti-CD206 (ab64693, Abcam; 1:1000), or normal rabbit IgG (2729, Cell Signaling Technology, Danvers, MA, USA) at 4 °C for 12 h. Following the washing with PBS, the macrophages were subjected to goat Alexa-488-labeled anti-mouse IgG (A-11001, Invitrogen, Waltham, MA, USA) or donkey Alexa-488-labeld anti-rabbit IgG (A-21206, Invitrogen, Waltham, MA, USA) secondary antibodies for 1 h and counterstained with DAPI (Nacalai Tesque, Kyoto, Japan). A BZ-9000 fluorescence microscope (Keyence Corporation, Itasca, IL, USA) was used to obtain images.

### 2.8. Statistical Analysis

All data were obtained from more than three independent experiments and were presented as mean ± SD. The statistical analysis was performed by a one-way ANOVA, followed by the Bonferroni post hoc test. The normality of the data distribution was validated by the Shapiro–Wilk normality test before performing the one-way ANOVA analysis. The definition of the statistical significance was a *p*-Value < 0.05.

## 3. Results

### 3.1. Emergence of the M1 Macrophages in the Rat Dental Pulp Tissue after the Direct Pulp Capping

**Figure 1:**
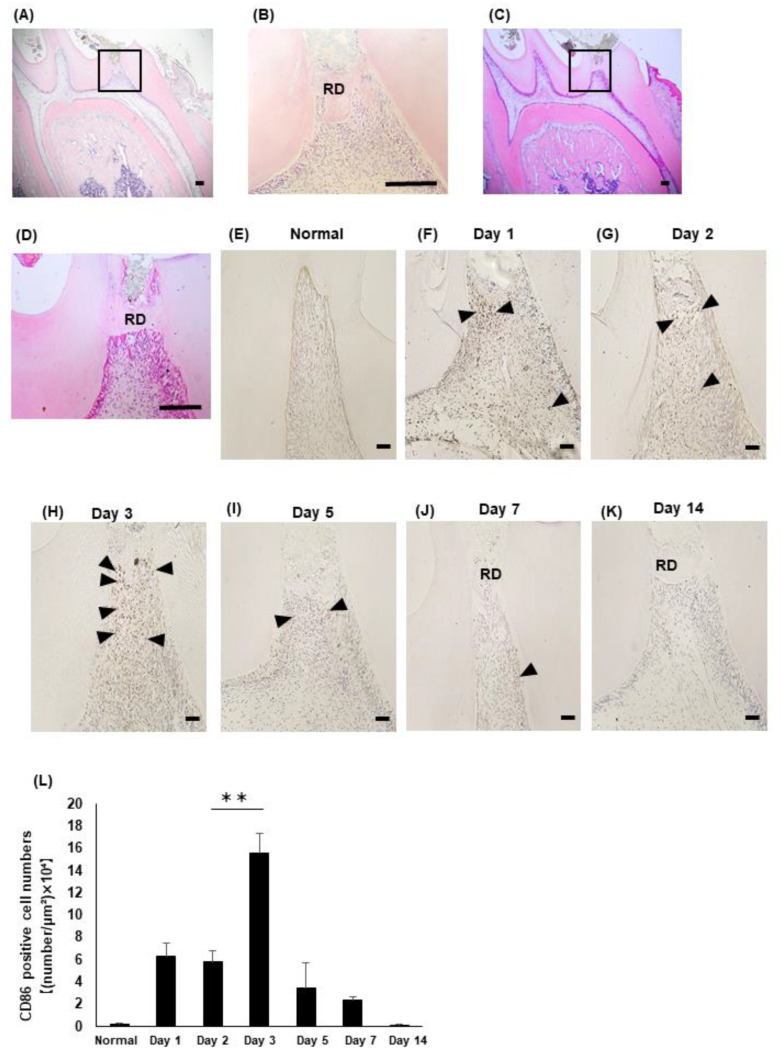
Emergence of CD86 in the rat dental pulp tissue after direct pulp capping. (**A**–**D**) Hematoxylin-eosin staining of the rat dental pulp tissue after 7 days (**A**,**B**), and 14 days (**C**,**D**) of treatment. (**B**,**D**) Higher magnification views of the boxed area in (**A**,**C**), respectively. (**E**–**K**) Immunohistochemical staining of CD86 in the normal dental pulp tissue (Normal) (**E**) and dental pulp tissue at 1 (**F**), 2 (**G**), 3 (**H**), 5 (**I**), 7 (**J**), and 14 days (**K**) post-operation. (**L**) The number of CD86-immunopositive M1 macrophages was quantified. N = 3, bars = 50 μm. RD: reparative dentin; arrow heads: CD86-immunopositive macrophages. ** *p* < 0.01.

The H-E staining revealed the induction of reparative dentin, but the exposure site had not closed at seven days after treatment (Figure 1A,B). At 14 days after treatment, the MTA induced a dense reparative dentin that covered the exposure site (Figure 1C,D). the immunohistochemical staining revealed that few CD86-positive M1 macrophages had emerged in the normal rat dental pulp tissue (Figure 1E). At one and two days after treatment, the emergence of the CD86-positive M1 macrophages increased in the dental pulp tissue (Figure 1F,G). At three days after treatment, more intense staining of CD86 was detected, even beneath the pulp exposure site (Figure 1H). Then, the emergence of the CD86-positive M1 macrophages gradually decreased at five and seven days after treatment (Figure 1I,J) and showed the same level as the control at 14 days post-operation (Figure 1K,L).

### 3.2. Emergence of the M2 Macrophages in the Rat Dental Pulp Tissue after the Direct Pulp Capping

**Figure 2:**
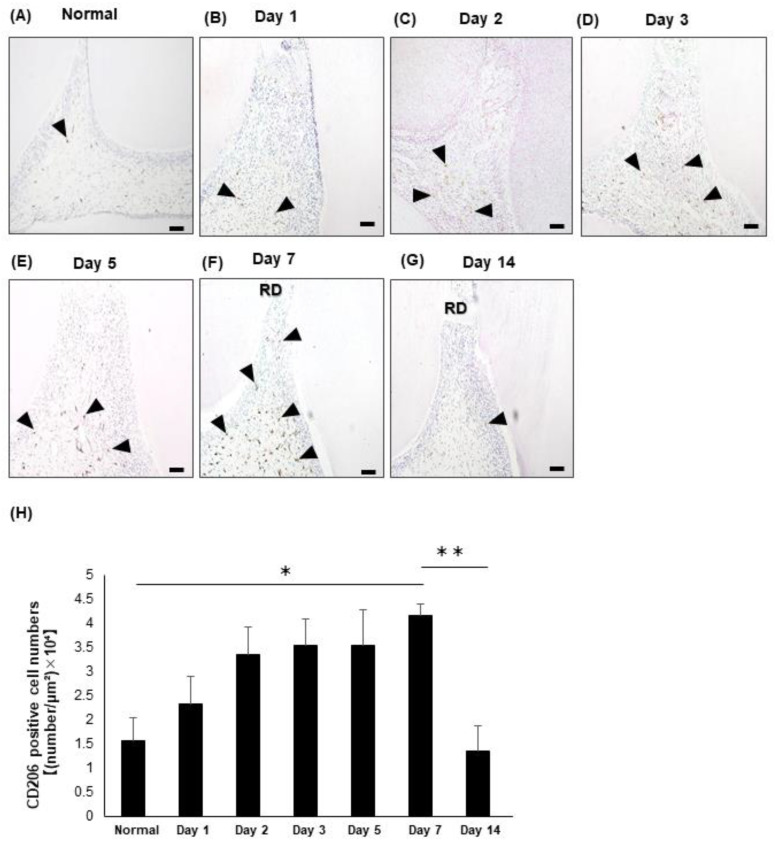
Emergence of CD206 in the rat dental pulp tissue after direct pulp capping. (**A**–**G**) Immunohistochemical staining of CD206 in the normal dental pulp tissue (Normal) (**A**) and dental pulp tissue at 1 (**B**), 2 (**C**), 3 (**D**), 5 (**E**), 7 (**F**), and 14 days (**G**) post-operation. (**H**) The number of CD206-immunopositive M2 macrophages was quantified. N = 3, bars = 50 μm. RD: reparative dentin; arrow heads: CD206-immunoreactive macrophages. * *p* < 0.05. ** *p* < 0.01.

The immunohistochemical staining revealed the presence of CD206-positive M2 macrophages in the normal dental pulp tissue (Figure 2A). At one day after treatment, the emergence of the CD206-positive M2 macrophages had slightly increased (Figure 2B). Then, the emergence of the CD206-positive M2 macrophages in the dental pulp tissue gradually increased at 2 (Figure 2C), 3 (Figure 2D) and 5 days (Figure 2E) after treatment. More intense staining of CD206 was detected in the dental pulp tissue even beneath the pulp exposure site at 7days after treatment (Figure 2F). At 14 days post-treatment, the reparative dentin covering the pulp exposure site has been induced and the emergence of the CD206-positive M2 macrophages had decreased to the same level as that of the control (Figure 2G,H).

### 3.3. Effects of the Macrophages on the Odontoblastic Differentiation of the DPSC Clones

**Figure 3:**
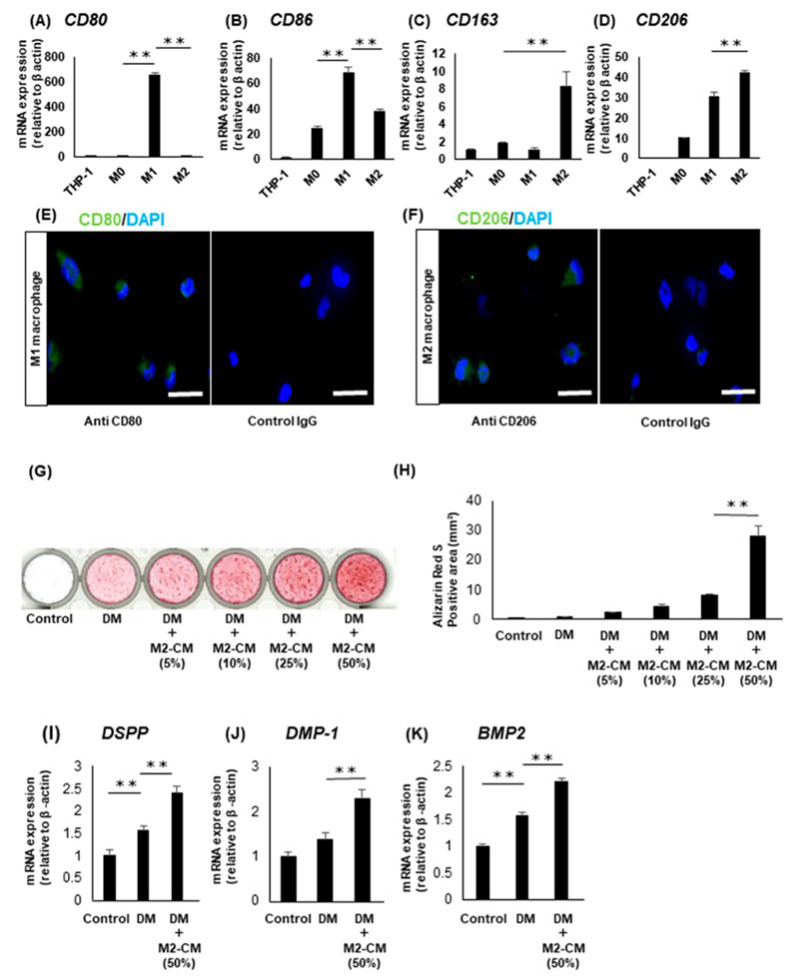
Effects of the M2 macrophages on the odontoblastic differentiation of the DPSC clones. (**A**–**D**) The expression of CD80 (**A**), CD86 (**B**), CD163 (**C**), and CD206 (**D**) in the THP-1 cells, M0, M1, and M2 macrophages. (**E**,**F**) Immunocytochemical staining of CD80 (**E**) in the M1 macrophages and CD206 (**F**) in the M2 macrophages. N = 3, bars = 50 μm. (**G**) Alizarin Red S staining images of the DPSC clones cultured in 10% FBS/α-MEM (Control) or differentiation medium (DM) with or without M2-CM for four weeks. (**H**) The quantification of the Alizarin Red S-positive area. (**I**–**K**) Gene expression of DSPP (**I**), DMP-1 (**J**), and BMP2 (**K**) in DPSC clones cultured in the Control, or DM with or without M2-CM for five days. Data are all presented as mean ± standard deviation (n = 4). ** *p* < 0.01.

The quantitative RT-PCR clarified that the M1 macrophages that were induced from THP-1 showed a higher expression of the M1 macrophage-related markers, CD80 and CD86, than those of the others (Figure 3A,B). However, the M2 macrophages induced from THP-1 showed the higher expression of the M2 macrophage-related markers, CD163 and CD206, than those of the others (Figure 3C,D). Additionally, the immunocytochemical staining revealed the protein expression of CD80 in the M1 macrophages (Figure 3E) and CD206 in the M2 macrophages (Figure 3F).

For mineralization assay, M2-CM increased the mineralization of the DPSC clones in a dose-dependent manner, compared with the DPSC clones cultured in CM or DM (Figure 3G,H). According to the results of the Alizarin Red S staining, 50% M2-CM showed the most upregulated effect on the mineralization of the DPSC clones, we decided to use this concentration of M2-CM in the following experiments. Next, we examined the effects of M2-CM on the expression of the odontoblast-related markers, DSPP, DMP-1, and BMP2 in the DPSC clones. The quantitative RT-PCR revealed that the DPSC clones supplemented with M2-CM, showed higher expression levels of these genes than those of the others (Figure 3I–K).

## 4. Discussion

Following a tissue injury, newly recruited or resident macrophage progenitors polarize to the M1/M2 direction in response to local stimuli [25,26]. Previous studies revealed that the M1 polarization is induced by LPS and IFN-γ [27] while the M2 polarization is induced by IL-4 and IL-13 [28]. In the case of severe inflammation or infection, M1 macrophages release TNF-α, IL-1β, IL-12, and IL-23 to participate in the immune response to stimuli [29]. Although a prolonged M1 phase is reported to cause tissue damage [30], the M1 to M2 polarization that reflect the macrophage phenotype change occur in an appropriate manner for successful wound healing [31]. In the present study, M1 macrophages increased soon after the dental pulp exposure and decreased as the M2 macrophages started to emerge, indicating an inflammatory response at the early phase of the wound healing by the M1 macrophages and their proper polarization to the M2 macrophages. In contrast with the M1 macrophages, the M2 macrophages show immunosuppressive effects by secreting IL-10 and TGF-β, inducing tissue repair, remodeling, and maintenance. In general, the M2 macrophages are known to further subdivide into M2a, M2b, M2c, and M2d macrophages, according to their biological function [32]. M2a macrophages increase cell growth and tissue repair [33], and M2b macrophages are known to regulate the depth of the immune response or immune reaction [34]. M2c macrophages, which are known as inactivated macrophages, are involved in the phagocytosis of apoptotic cells [35]. M2d macrophages have been reported to promote angiogenesis and tumorigenesis [36]. In addition, CD206, which is known as mannose receptor C type 1 [37], is reported to be expressed in M2a and M2c macrophages [38]. Thus, the subtype of M2 macrophages involved in the regenerative event after a dental pulp exposure is thought to be mainly the M2a macrophage subtype. In the present study, the number of M1 macrophages increased at the pulp exposure site soon after injury, followed by an increase in the M2 macrophages as M1 macrophages disappeared and the reparative dentin formation started. These results suggest that M1 macrophages increase in response to the pulp exposure, and M2 macrophages play a role in the reparative dentin formation, indicating their possible contribution to injury repair.

During the injury repair of dental pulp tissue, the process of the odontoblastic differentiation of DPSCs is essential for synthesizing the reparative dentin at the pulp exposure site. MTA has been reported to release calcium ions [39,40] and induce reparative dentin after the pulp exposure when it is applied as a direct pulp capping material [41,42,43,44]. We previously reported that CaCl_2_ induces mineralization and odontoblastic differentiation of dental pulp cells [45]. As for the M2 macrophages, recent studies reported that M2 macrophages induce mineralization of the mesenchymal stem cells [46,47]. In the present study, the CaCl_2_ treatment induced an odontoblast-like differentiation of the DPSC clones, and these upregulated effects were significantly enhanced by the conditioned medium from the M2 macrophages. These results suggest that the M2 macrophages might secret factors that can enhance the odontoblastic differentiation of DPSCs. Although the critical components included in M2-CM that induced the odontoblastic differentiation of the DPSC clones remain unclear, further investigation of factors from the M2 macrophages, such as RNA, proteins, and lipids that are included in exosomes and microvesicles, would be helpful to clarify the underlying mechanism of the odontoblastic differentiation in the process of reparative dentin formation. Additionally, the rigorous assessment of their ability to induce reparative dentin after pulp exposure could contribute to develop a pulp capping material that can facilitate wound healing after a pulp injury.

## 5. Conclusions

We verified the detailed expression pattern of M1 and M2 macrophages after a direct pulp capping treatment. Additionally, we validated the promotive effects of the M2 macrophages on the odontoblastic differentiation of the DPSC clones. Further clarification of the immune response after a pulp exposure will be helpful to understand the precise mechanisms of injury repair of dental pulp tissue. Moreover, identification of factors from M2 macrophages that can induce reparative dentin formation as well as the odontoblastic differentiation of DPSCs would be helpful to develop a novel method for vital pulp therapy.

## Data Availability

Not applicable.
